# Associations between second to fourth digit ratio, cortisol, vitamin D, and body composition among Polish children

**DOI:** 10.1038/s41598-021-86521-7

**Published:** 2021-03-29

**Authors:** Paulina Pruszkowska-Przybylska, Aneta Sitek, Iwona Rosset, Marta Sobalska-Kwapis, Marcin Słomka, Dominik Strapagiel, Elżbieta Żądzińska, Niels Morling

**Affiliations:** 1 Department of Anthropology, Faculty of Biology and Environmental Protection, University of Lodz, 90-237 Lodz, Poland; 2 Biological Anthropology and Comparative Anatomy Research Unit, School of Medicine, The University of Adelaide, Adelaide, SA 5005 Australia; 3 The Biobank Lab, Department of Molecular Biophysics, Faculty of Biology and Environmental Protection, University of Lodz, Lodz, Poland; 4 BBMRI.Pl Consortium, Wrocław, Poland; 5 Section of Forensic Genetics, Department of Forensic Medicine, Faculty of Health and Medical Sciences, University of Copenhagen, Frederik V’s Vej 11, 2100 Copenhagen, Denmark

**Keywords:** Biochemistry, Health care, Medical research

## Abstract

Associations between body characteristics (body composition: fat mass, muscle mass, cell, and water mass as well as body proportion—BMI), the 2D:4D digit ratio, and the concentrations of cortisol and vitamin (25-OH)D among Polish children have not been studied before. A total of 133 (73 girls and 60 boys) healthy Polish children aged 7–11 years were examined. The investigation was divided into three parts: measuring (the length of the second and fourth fingers in both hands, body composition, and body height and mass), questionnaires (socioeconomic status), and laboratory investigations (25-OH vitamin D and cortisol concentrations in saliva measured with ELISA methods). Boys with digit ratios below 1 had lower vitamin D concentration than those with digit ratios equal to or higher than 1 (Z = − 2.33; p = 0.019). Only boys with the male-typical pattern of 2D:4D digit ratio tended to have a lower 25-OH vitamin D concentration in saliva. Thus, it might indicate an effect of prenatal programming on the concentrations of steroid hormones in later life. Neither vitamin D, 2D:4D digit ratio nor the cortisol level was associated with the body components or proportions. More studies are needed to evaluate the molecular and genetic background of this phenomenon.

## Introduction

The 2D:4D digit ratio is a widely used indicator of the proportion of prenatal sex hormones—testosterone and oestrogen. Due to the results of the study conducted by Lutchmaya et al. which showed a correlation between testosterone to oestradiol ratio and 2D:4D (right hand) there was susception that a low digit ratio is associated with a high prenatal exposition to testosterone, and inversely, a high digit ratio is associated with a high exposition to prenatal oestrogen^1^. Sex hormones regulate the expression of the genes involved in chondrocyte proliferation affecting the finger length during the prenatal period^2^. Rodent studies have shown that there are more androgen and oestrogen receptors on the fourth than the second finger^2,3^. Thus, the 2D:4D proportion depends on the length of the fourth finger. Increased levels of prenatal testosterone are associated with increased length of the fourth finger, and increased levels of prenatal oestrogen are linked with decreased length of the fourth finger among mice^2–5^. However, Huber et al.^6^ tried to replicate these influential findings and found essentially the opposite pattern of results, which presented that the 2D:4D ratio pattern in both sex among mice is not developed in prenatal period. Additionally, some human studies did not show that fetal or/and maternal sex hormones are associated with the 2D:4D ratio of the offspring^7–9^. Richards et al.^10^ repeated the study of Lutchmaya et al. They found no statistically significant association between the digit length and the levels of the second-trimester sex hormones. In another study, Richards et al.^11^ presented meta-analyses of the digit ratio (2D:4D) and the congenital adrenal hyperplasia (CAH), which showed that the effect sizes were ~ 50% smaller than those of an earlier meta-analysis^12^.

Beside the statement of Manning et al., which suggested that the 2D:4D digit ratio indicates sex hormones proportions in later life^13^, the more recent studies (e.g., Kowal et al.^14^) do not support this hypothesis. Zhang et al.^15^ and Hönekopp et al.^16^ also showed with meta-analyses that the current testosterone levels in saliva and serum are not statistically significantly associated with digit ratio.

Cortisol, vitamin D, and sex hormones are important molecules, which are all derived from cholesterol, regulate various metabolic networks^17–19^, and may affect the body composition at the various stages of human ontogenesis^20–22^. In the current study, we evaluated if vitamin D and cortisol are associated with the 2D:4D finger ratio and other phenotypic traits such as body composition. There is some evidence of their interaction, but the association with the 2D:4D ratio has not been thoroughly explored in previous studies.

To the best of our knowledge, the relationship between the 2D:4D ratio and vitamin D level has never been investigated. Some studies indicate a relation between vitamin D and testosterone levels. Pilz et al.^23^ showed that increased vitamin D levels might indicate higher testosterone levels. However, the findings were not consistent. According to Heijboer et al.^24^ and Lerchbaum et al.^25^, vitamin D supplementation was not associated with an increase in testosterone level.

Cortisol, vitamin D, and prenatal sex hormones in children have been examined in the past, but the parameters have never been studied simultaneously. The association between 2D:4D and cortisol was not statistically significant in a study of healthy adult males^26^. On the other hand, two studies showed opposite findings: Liening et al.^27^ underlined that the cortisol level is positively corelated with the testosterone level. Additionally, hormonal and behavioural studies conducted by Portnoy et al.^28^ presented that in males, low cortisol reactivity was associated with low 2D:4D. Thus, here comes the question if cortisol might be associated with the 2D:4D digit ratio. There are also studies that suggest vitamin D may affect cortisol level. Al-Dujaili et al.^29^ showed that vitamin D supplementation may reduce cortisol level.

As we presented above vitamin D may affect testosterone and cortisol concentrations. Most likely, this is linked with the function of the vitamin D receptor (VDR), which is present in Leydig cells in the testes that produce testosterone^30^, but also in the hypothalamus regulating activation of the adrenal gland to produce cortisol^31^. The simultaneous effect of vitamin D, cortisol, and testosterone concentration was tested by Crewther et al.^32^. They showed that only among low-25(OH)D individuals with increased cortisol concentration reactivity to testosterone was weaker. Additionally, Kowal et al. reported a positive correlation between testosterone and cortisol levels, but their relation to 2D:4D ratio was statistically nonsignificant^14^. There are also some findings in the case of vitamin D, cortisol, 2D:4D digit ratio, and body composition or BMI^14,22,33^, but all of these variables have never been analysed simultaneously.

The aim of the current exploratory study was to evaluate associations between cortisol, 25(OH) vitamin D, and the 2D:4D digit ratio. We also investigated if there is an association between cortisol concentration and body composition and BMI.

## Results

**Figure 1:**
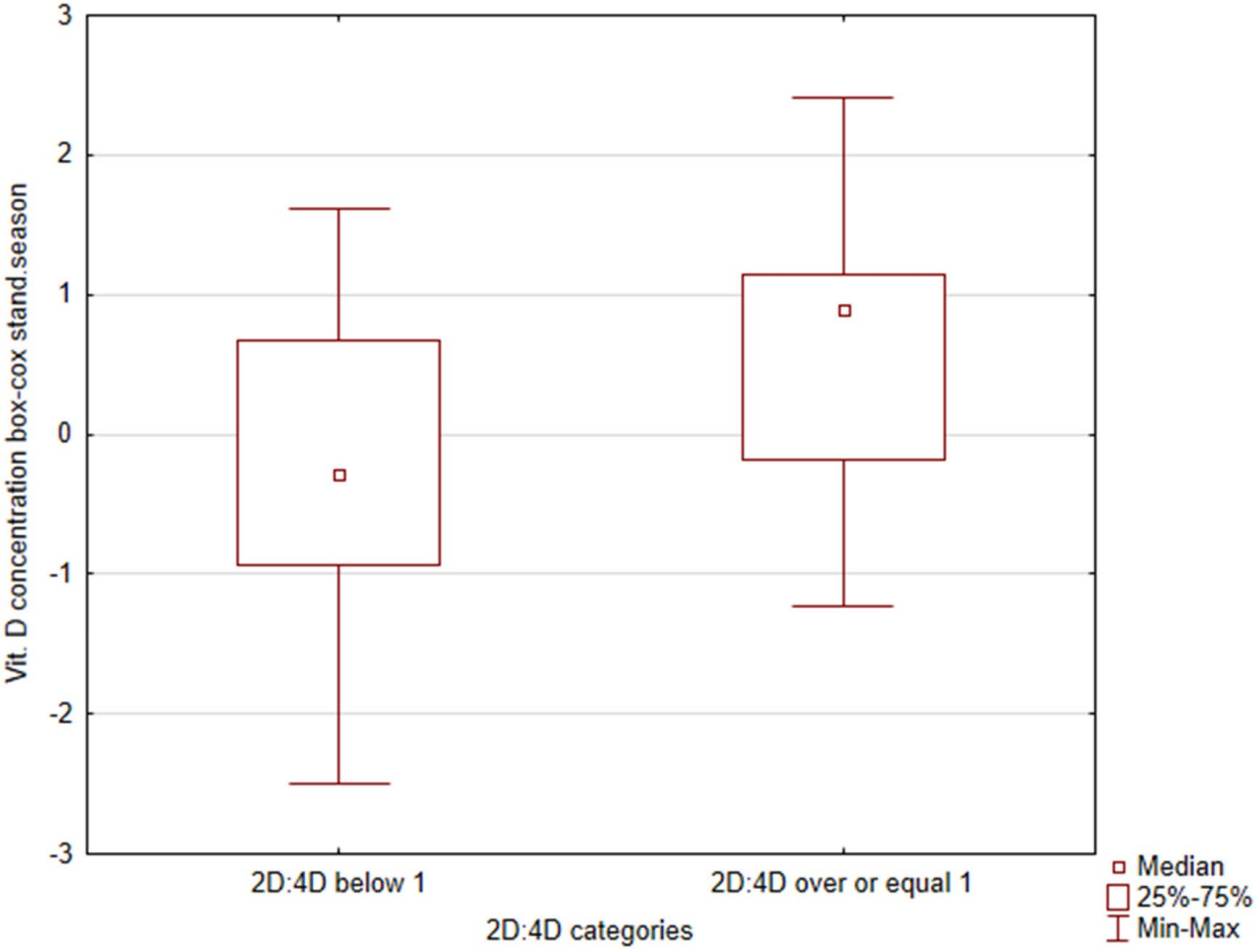
Difference in vitamin D concentration between boys with 2D:4D < 1 or ≥ 1.

Boys with digit ratio values below 1 (male pattern) had lower vitamin D concentration (median =  − 0.29) than those with digit ratio equal to or higher than 1 (female pattern) (median = 0.89) (Z = − 2.33; p = 0.019) (Table 1; Fig. 1). There were no statistically significant correlations between vitamin D and cortisol concentrations, 2D:4D ratio, and body composition (Table 2).

**Table 1:** The 2D:4D digit ratio pattern, cortisol and vitamin D concentrations in boys and girls.

	2D:4D digit ratio^a^	N	SD	Q1	Median	Q3	Z	p
	0	56	0.876	− 0.590	− 0.274	− 0.034	− 1.194	0.2324
	1	17	0.819	− 0.392	− 0.170	0.335		
Boys	0	46	1.020	− 0.557	− 0.314	0.207	− 1.250	0.2114
	1	14	0.788	− 0.361	0.145	0.723		
**Vit. D concentration**								
Girls	0	56	1.045	− 0.823	− 0.203	0.785		
	1	17	0.969	− 0.599	0.096	0.877	0.646	0.5183
Boys	0	46	1.019	− 0.914	− 0.192	0.772		
	1	14	1.233	− 0.701	0.535	1.005	− 2.333	0.0196

^a^0: 2D:4D < 1; 1: 2D:4D ≥ 1.

**Table 2:** Spearman correlations between the investigated variable.

Variable	& Covariates	R	p value	R	p value
		Boys N = 60		Girls N = 73	
Vit. D concentration	Cortisol concentration	0.273	0.0345	0.159	0.1787
	FM%	− 0.021	0.8708	0.099	0.4054
	BCM%	0.286	0.0268	0.112	0.3443
	MM%	0.193	0.1387	− 0.028	0.8138
	TBW%	− 0.024	0.8550	− 0.105	0.3748
	BMI z-score	− 0.002	0.9899	0.186	0.1149
	2D:4D	0.186	0.1549	0.001	0.9907
	SES	0.146	0.2647	− 0.079	0.5049
Cortisol concentration	FM%	− 0.117	0.3723	− 0.091	0.4424
	BCM%	0.224	0.0856	0.038	0.7465
	MM%	0.237	0.0682	0.172	0.1447
	TBW%	0.061	0.6452	0.071	0.5504
	BMI z-score	− 0.146	0.2657	0.005	0.9674
	2D:4D	0.036	0.7823	0.132	0.2658
	SES	0.168	0.1986	0.104	0.3833
2D:4D digit ratio	FM%	0.077	0.5602	− 0.083	0.4851
	BCM%	0.155	0.2370	0.062	0.6049
	MM%	0.026	0.8461	0.152	0.1997
	TBW%	− 0.105	0.4227	0.152	0.1994
	BMI z-score	0.015	0.9070	0.085	0.4724
	SES	0.053	0.6903	0.222	0.0585

Bonferroni correction threshold of significance p < 0.002.

Multiple regression models designed for all predictor variables (FM%, MM%, BCM%, TBW%, and BMI), separately for boys and girls, included the following outcome variables: vitamin D concentration, cortisol concentration, 2D:4D digit ratio (right hand), standard of living (three categories), and parental education. None of the applied models were statistically significant (Table 3).

**Table 3:** Multiple regression models for all predictor variables (FM%, MM%, BCM%, TBW%, and BMI), separately for boys and girls, included the following outcoming variables: the vitamin D and cortisol concentration, 2D:4D digit ratio (right hand), the standard of living (three categories) and parental education.

	Girls N = 73								
	b*	Std.err. of b*	b	Std. err. of b	t(66)	p-value	R^2^	F	p
**FM%**									
Intercept			3.022	6.133	0.493	0.624	< 0.001	0.943	0.471
Cortisol concentration	− 0.089	0.12	− 0.1	0.136	− 0.74	0.462			
Vit. D concentration	0.086	0.12	0.088	0.122	0.722	0.473			
Parental education	− 0.142	0.138	− 0.149	0.146	− 1.025	0.309			
2D:4D digit ratio	− 0.072	0.124	− 3.618	6.185	− 0.585	0.561			
SES—high vs. low	0.296	0.305	0.575	0.593	0.969	0.336			
SES—medium vs. low	0.333	0.315	0.643	0.608	1.057	0.294			
**MM%**									
Intercept			− 4.617	6.142	− 0.752	0.455	0.018	1.223	0.306
Cortisol concentration	0.119	0.119	0.137	0.136	1.006	0.318			
Vit. D concentration	0.002	0.118	0.002	0.122	0.013	0.99			
Parental education	0.243	0.137	0.259	0.146	1.775	0.08			
2D:4D digit ratio	0.098	0.122	4.977	6.195	0.803	0.425			
SES—high vs. low	− 0.195	0.302	− 0.383	0.594	− 0.645	0.521			
SES—medium vs. low	− 0.151	0.311	− 0.297	0.609	− 0.487	0.628			
**BCM%**									
Intercept			− 3.262	6.379	− 0.511	0.611	< 0.001	0.375	0.892
Cortisol concentration	0.019	0.123	0.021	0.141	0.151	0.881			
Vit. D concentration	0.119	0.123	0.123	0.127	0.971	0.335			
Parental education	0.101	0.142	0.108	0.152	0.71	0.48			
2D:4D digit ratio	0.058	0.127	2.924	6.433	0.454	0.651			
SES—high vs. low	0.176	0.313	0.346	0.617	0.561	0.577			
SES—medium vs. low	0.213	0.322	0.417	0.633	0.66	0.512			
**TWB%**									
Intercept			− 5.858	5.982	− 0.979	0.331	0.031	1.389	0.232
Cortisol concentration	0.058	0.118	0.065	0.132	0.494	0.623			
Vit. D concentration	− 0.064	0.118	− 0.065	0.119	− 0.544	0.588			
Parental education	0.192	0.136	0.201	0.142	1.415	0.162			
2D:4D digit ratio	0.134	0.122	6.628	6.032	1.099	0.276			
SES—high vs. low	− 0.413	0.3	− 0.797	0.578	− 1.378	0.173			
SES—medium vs. low	− 0.399	0.309	− 0.765	0.593	− 1.29	0.202			
**BMI z-score**									
Intercept			− 3.068	5.97	− 0.514	0.609	0.128	1.611	0.158
Cortisol concentration	− 0.027	0.117	− 0.031	0.132	− 0.231	0.818			
Vit. D concentration	0.167	0.117	0.17	0.119	1.432	0.157			
Parental education	− 0.084	0.135	− 0.089	0.142	− 0.624	0.535			
2D:4D digit ratio	0.037	0.121	1.833	6.02	0.304	0.762			
SES—high vs. low	0.715	0.297	1.388	0.577	2.406	0.019			
SES—medium vs. low	0.656	0.306	1.268	0.592	2.141	0.036			

Boys N = 60									
**FM%**									
Intercept			− 6.312	5.262	− 1.2	0.236	< 0.001	0.802	0.573
Cortisol concentration	− 0.091	0.137	− 0.093	0.141	− 0.661	0.511			
Vit. D concentration	− 0.034	0.141	− 0.032	0.133	− 0.244	0.808			
Parental education	− 0.119	0.142	− 0.108	0.129	− 0.836	0.407			
2D:4D digit ratio	0.149	0.136	5.817	5.324	1.093	0.28			
SES—high vs. low	0.294	0.272	0.579	0.537	1.079	0.286			
SES—medium vs. low	0.332	0.273	0.664	0.546	1.216	0.229			
**MM%**									
Intercept			4.144	5.202	0.797	0.429	< 0.001	0.967	0.456
Cortisol concentration	0.12	0.136	0.123	0.139	0.879	0.384			
Vit. D concentration	0.116	0.14	0.109	0.131	0.833	0.409			
Parental education	0.1	0.141	0.091	0.128	0.712	0.479			
2D:4D digit ratio	− 0.095	0.135	− 3.687	5.263	− 0.701	0.487			
SES—high vs. low	− 0.231	0.27	− 0.455	0.531	− 0.857	0.395			
SES—medium vs. low	− 0.329	0.271	− 0.656	0.54	− 1.215	0.23			
**BCM%**									
Intercept			− 0.767	5.131	− 0.15	0.882	< 0.001	0.818	0.561
Cortisol concentration	0.056	0.137	0.056	0.138	0.41	0.684			
Vit. D concentration	0.207	0.141	0.191	0.13	1.473	0.147			
Parental education	− 0.056	0.142	− 0.05	0.126	− 0.397	0.693			
2D:4D digit ratio	0.017	0.136	0.635	5.19	0.122	0.903			
SES—high vs. low	0.135	0.272	0.259	0.524	0.495	0.623			
SES—medium vs. low	− 0.016	0.273	− 0.03	0.533	− 0.057	0.955			
**TBW%**									
Intercept			6.144	5.234	1.174	0.246	< 0.001	0.941	0.474
Cortisol concentration	0.016	0.136	0.016	0.14	0.117	0.907			
Vit. D concentration	0.003	0.14	0.003	0.132	0.021	0.983			
Parental education	0.206	0.141	0.188	0.129	1.457	0.151			
2D:4D digit ratio	− 0.143	0.135	− 5.615	5.295	− 1.06	0.294			
SES—high vs. low	− 0.318	0.27	− 0.629	0.534	− 1.177	0.244			
SES—medium vs. low	− 0.333	0.271	− 0.667	0.543	− 1.228	0.225			
**BMI z-score**									
Intercept			0.384	5.125	0.075	0.941	0.033	1.33	0.26
Cortisol concentration	− 0.148	0.134	− 0.152	0.137	− 1.107	0.273			
Vit. D concentration	− 0.02	0.137	− 0.019	0.13	− 0.144	0.886			
Parental education	− 0.305	0.138	− 0.278	0.126	− 2.205	0.032			
2D:4D digit ratio	− 0.019	0.133	− 0.731	5.185	− 0.141	0.888			
SES—high vs. low	0.26	0.265	0.512	0.523	0.98	0.332			
SES—medium vs. low	0.133	0.266	0.266	0.532	0.5	0.619			

Bonferroni correction: threshold of significance p < 0.008.

## Discussion

Our study was the first attempt to simultaneously investigate vitamin D, cortisol, 2D:4D and body composition in Polish children. We demonstrated that 2D:4D was not correlated with the concentration of vitamin D or cortisol when treated as a continuous variable. Moreover, the body components were not correlated with the concentrations of the investigated steroids or the 2D:4D digit ratio. There was only one statistically significant observation, which showed that boys with male-typical patterns of digit ratio (2D:4D ratio lower than 1) might have decreased vitamin D concentrations. However, taking into account the limitations such as a lack of statistical significance in the simultaneous examination of the four comparisons (the 2D:4D ratio vs. the cortisol concentration in males and females and the 2D:4D ratio vs. the vitamin D in males and females) as well as the small size of the investigated male groups (n = 46 and n = 14 for 2D:4D < 1 and 2D:4D > 1, respectively), this analysis can be considered underpowered, and its outcome shall not be used as a reference to draw strong conclusions unless a larger study confirms the result.

Fitzgerald et al.^34^ found a weak negative association between the concentration of vitamin D and testosterone among boys aged 15–18 years, while Pilz et al.^23^ suggested a positive association, with vitamin D supplementation increasing the testosterone level in men. It has been suggested that the association between testosterone and vitamin D concentrations is caused by the vitamin D receptors (VDR) in Leydig cells in the testis that produce testosterone^29^. Our opposite findings might be a result of the prepubertal status of the young boys (7–11 years) in our study sample. The vitamin D level among pre-pubertal boys may not per se be directly associated with the current testosterone concentration due to premature testis without fully developed vitamin D receptors. Of note, Heijboer et al.^24^ and Lerchbaum et al.^25^ showed that vitamin D supplementation was not linked with an increase in testosterone levels. Our research is the first to examined for an association between the 2D:4D digit ratio and vitamin D concentration. The lack of statistically significant results might stem from a weak association between 2D:4D and the current concentration of sex hormones that was proposed in some studies^12,15^ and the small number of investigated individuals.

In a similar manner to our investigation, Wrzosek et al.^35^ did not find any association between vitamin D and cortisol concentration. Our results are not in line with studies which suggested that cortisol inhibits intestinal calcium transport causing enhanced production of endogenous vitamin D^36^. On the other hand, Fitzgerald et al.^34^ showed that adolescent ice hockey players, who had normal concentrations of vitamin D, had decreased levels of cortisol. Additionally, Al-Dujaili et al.^29^ showed that vitamin D supplementation may reduce the cortisol level. Wakefield et al.^37^ showed that chronic maternal stress during pregnancy was negatively associated with vitamin D and cortisol levels in postnatal life. It seems likely that the associations between vitamin D and cortisol and corticotropin-releasing hormone (CRH) might be changed under stressful events.

We did not observe any statistically significant association between the 2D:4D ratio and cortisol concentration as observed by Ribeiro et al.^26^ and Kowal et al.^14^ in men. In contrast to this, Liening et al.^27^ found that the level of cortisol is positively associated with that of testosterone and, thus it might be linked with 2D:4D digit ratio. Similarly, Portnoy et al.^28^ reported that a low cortisol reactivity was associated with low 2D:4D in males.

Our investigation of the association between the concentration of steroids and the body components showed no statistically significant results. The impact of cortisol on particularly the fat and muscle mass among children is unclear. Studies have shown positive associations between the cortisol concentration and the amount of fat tissue among children^38,39^, while other studies have pointed at an inverse association^20,40^. We also showed that, similarly as in our previous study^22^, the vitamin D concentration was not associated with the majority of body components. Hence, vitamin D may be important by triggering calcium absorption leading to hormonal equilibrium that results in balanced nutrition of the body. There is no certain evidence of an association between vitamin D concentration and body composition, thus more studies are needed in this area.

Direct associations between the 2D:4D digit ratio and body components were previously reported, e.g., by Pruszkowska-Przybylska et al.^33^, who found the relationship among 6–13-year girls between a male pattern of the 2D:4D ratio and increased muscle mass. However, in our current investigations, after including the 25(OH)D and cortisol concentrations in the regression model, no statistically significant association that could explain the variability of the body components was found.

## Limitations

The number of investigated individuals in the various groups was not large enough to reach firm conclusions based on robust statistical power. Thus, further studies of larger groups of individuals are needed. Furthermore, we would like to underline that more and more studies have pointed to questionable credibility of 2D:4D finger ratio as an indicator of prenatal sex hormone exposure^41^. Our findings regarding 2D:4D and vitamin D level had weak statistical power, and so more studies which verify the genetic and biochemical background of this phenomenon are needed. Additionally, the Immunoassay (ELISA) may be a less precise method to assess vitamin D and cortisol concentrations than other methods such as mass spectrometry^42^. However, the ELISA method and similar chemiluminescence immunoassays are still very useful for measuring the total 25(OH)D concentration^14,43,44^.

## Conclusions

The results indicated that only boys aged 7–11 years with a 2D:4D digit ratio lower than 1 had slightly decreased vitamin (25-OH)D concentrations in the saliva than those with a 2D:4D ratio at 1 or higher. However, neither vitamin D, 2D:4D digit ratio nor the cortisol level was associated with the body components or proportions.

## Methods

A total of 133 (73 girls and 60 boys) healthy individuals aged 7–11 years (mean = 8.92; SD = 1.58) were included in the current study. Participants were randomly selected in primary schools in central Poland (Lodz, a city with approximately 700,000 inhabitants).

The investigation was divided into three parts: body measuring, questionnaires, and laboratory investigations. The measuring part consisted of: anthropometric measurements of body height (cm), body mass (kg), and the length of the second and fourth fingers (mm) of the left and right hands; body composition measurements using the BIA-method (MM%—muscle mass; FM%—fat mass; BCM%—body cellular mass; TBW%—total body water) using BIA (BIA-101 ASE, Akern, Italy). The BIA method is based on the measurement of the electrical impedance in body tissues. The results are calculated as the sum of the geometric resistance (active resistance) and reactance (passive resistance)^45^. We used Plethysmograph with 4 foil-coated electrodes placed in the midline of the dorsal surface of the hands and feet. Measurements were performed with the participants in supine position, which helps to stabilize and balance the body level fluids^46^.

The questionnaires included questions about parental education and standard of living due to their significant role in affecting body composition and proportions^22,47^. Parental education was divided into three categories: (1) basic or vocational education (8 years at obligatory primary school plus 3 years at vocational school); (2) secondary education (4–5 years at secondary school) or bachelor's degree (3 years of education after secondary school); and (3) higher education (full university degree—Master of Science degree). Standard of living was defined according to the parents’ declaration as followed: (1) low standard of living (we live very poorly, we have insufficient resources for basic needs or we live modestly, we have to be very economical; (2) medium standard of living (we live on average, it is enough for us every day, but we have to save on more serious purchases); and (3) high standard of living (we live well enough for us without many special savings or we live very well—we can afford full luxury).

The laboratory part included investigations of the vitamin D and cortisol concentrations in saliva using the enzyme-linked immunosorbent assay (ELISA) method. At least 5 ml of saliva was collected between 8 am and 2 pm using falcon-tubes (Nestbiotechnology). Eating and chewing gum were forbidden for at least 30 min before saliva collection took place. Immediately after sampling, the saliva samples were stored at 2–8 °C, and, thereafter, stored at − 20 °C until investigation. Just before testing, the samples were thawed and centrifuged at 2000*g*.

The ELISA kits were used to measure the cortisol and 25(OH)D concentrations, respectively, with the DRG Salivary Cortisol ELISA (SLV-2930, USA kit), and Human Vitamin D(VD)ELISA Kit (Sun-Red Biotechnology Company). The microplate reader (SpectraMax i3, Molecular Devices) measuring the absorbance at 450 nm was used to assess the concentration of the investigated molecules. The 4PL method standard curves were created using open-source software (https://www.aatbio.com/tools/four-parameter-logistic-4pl-curve-regression-online-calculator/). All results included in the analysis were within the assay range according to the information supplied by the kit-producers. The intra-assay CVs were 8.22% for vitamin D and 7.89% for cortisol concentration.

This current study was conducted according to the guidelines of the Declaration of Helsinki, and all procedures involving research study participants were approved by the Ethical Commission at the University of Lodz (nr 19/KBBN-UŁ/II/2016). Written informed consent was obtained from all parents of the children.

### Statistical analysis

The z-score values for the FM%, MM%, BCM%, TBW%, and BMI were calculated and standardised for calendar age (years) and sex.

The Wilcoxon signed-rank test showed that there was no statistically significant difference between the 2D:4D ratios of the left and right hands among all participants (T = 3122.500; Z = 0.281; p = 0.779). Because some of the studies suggest that R2D:4D has a larger sex difference and therefore might be more sensitive to fluctuations in early hormone levels (e.g.^12^) only measurements of the right hand were included for further analyses.

Due to the skewness of the distributions for cortisol and vitamin D concentrations, the Box-Cox transformation was applied. Because the saliva samples were collected in two time periods, Autumn (November–December) and Spring (June), the vitamin D concentration was standardised on the season of collection due to seasonal variation of vitamin D. Due to a daily variation of the cortisol concentration in all performed calculations, the cortisol concentration was standardised by the hour of the day at which the sample was collected.

The Mann–Whitney U test was applied to examine for differences in the vitamin D and cortisol concentrations. The digit ratio was treated as a binary (0/1) variable (0: 2D:4D < 1; 1: 2D:4D ≥ 1).

The Spearman test was used to evaluate correlations between all continuous variables. Due to the multiple comparisons, we calculated a new alpha for the statistical significance using the Bonferroni correction (alpha/number of comparison). Therefore, the adjusted threshold of significance was 0.05/21 = 0.002.

Multiple regression models were used to examine how outcome variables explain variability of the body composition (FM%, MM%, BCM%, TBW%) and BMI. We included five outcome variables: vitamin D, cortisol, 2D:4D digit ratio of the right hand, standard of living, and parental education. Due to the multiple comparisons, we adjusted the alpha value for significance using the Bonferroni correction. Therefore, the adjusted threshold of significance was 0.05/6 = 0.008.
